# Diet in Fever

**Published:** 1888-10

**Authors:** T. R. Chalmers


### DIET IN FEVER.

BY DR. T. R. CHALMERS.

In all fevers, which are classed together as being apparently due to a poison in the blood, the art of diet consists in giving an almost continuous supply of nutriment, holding very soluble aliments in a diluted form. There is nothing so digestible as water, and we take advantage of this high digestibility to get whatever it can dissolve, digested along with it. For the first three or four days, patients previously strong, should have only farinaceous food, well boiled and cooled to the temperature of the body.

Evidence has been already produced of the power which oatmeal possesses of sustaining force under the trying circumstances of toil. Now, fever closely resembles muscular effort in its arrest of the digestive functions, at the same moment that it makes an urgent demand for nutriment. We know by the quantities of urea and phosphates in the urine, and by the fæcal excretion, that the muscles and nerves of the bed-ridden sufferer are melting away as fast as if he were scaling the Alps with nothing to eat.

It is quite reasonable to transfer the experiences derived from health to sickness, and to feel satisfied that we are not wasting previous opportunities when we are giving fever patients such a time honored diet as oatmeal gruel, care being taken that it is thoroughly boiled. After three days the tissues are beginning to suffer, and it is advisable to add chicken broth, meat jelly and strong soup. Let that be supplied which the emaciation shows to be passing away—nitrogenous tissue.

